# Time needed to intubate and suction a manikin prior to instituting positive pressure ventilation: a simulation trial

**DOI:** 10.1007/s00431-020-03759-5

**Published:** 2020-08-04

**Authors:** Francesco Cavallin, Giulia Res, Chiara Monfredini, Nicoletta Doglioni, Paolo Ernesto Villani, Gary Weiner, Daniele Trevisanuto

**Affiliations:** 1 Solagna, Italy; 2 Department of Women and Children Health, University of Padova, Via Giustiniani, 3, 35128 Padova, Italy; 3 Fondazione Poliambulanza, Brescia, Italy; 4 Division of Neonatal-Perinatal Medicine, Department of Pediatrics and Communicable Diseases, University of Michigan, Ann Arbor, MI USA

**Keywords:** Intubation, Manikin, Meconium aspiration syndrome, Neonate, Suctioning, Time

## Abstract

Tracheal suctioning in non-vigorous newborn delivered through meconium-stained amniotic fluid (MSAF) is supposed to delay initiation of positive pressure ventilation (PPV), but the magnitude of such delay is unknown. To compare the time of PPV initiation when performing immediate laryngoscopy with intubation and suctioning vs. performing immediate PPV without intubation in a manikin model. Randomized controlled crossover (AB/BA) trial comparing PPV initiation with or without endotracheal suctioning in a manikin model of non-vigorous neonates born through MSAF. Participants were 20 neonatologists and 20 pediatric residents trained in advanced airway management. Timing of PPV initiation was longer with vs. without endotracheal suctioning in both pediatric residents (mean difference 13 s, 95% confidence interval 8 to 18 s; *p* < 0.0001) and neonatologists (mean difference 12 s, 95% confidence interval 8 to 16 s; *p* < 0.0001). The difference in timing of PPV initiation was similar between pediatric residents and neonatologists (mean difference − 1 s, 95% confidence interval − 7 to 6 s; *p* = 0.85).

*Conclusions*: Performing immediate laryngoscopy with intubation and suctioning was associated with longer—but not clinically relevant—time of initiation of PPV compared with immediate PPV without intubation in a manikin model. While suggesting negligible delay in starting PPV, further studies in a clinical setting are warranted.

*Registration*: clinicaltrial.gov NCT04076189.

**Table:** 

**What is Known:** *• Management of the non-vigorous newborn delivered through meconium-stained amniotic fluid remains still controversial.* *• Tracheal suctioning in non-vigorous newborn delivered through meconium-stained amniotic fluid is supposed to delay initiation of positive pressure ventilation, but the magnitude of such delay is unknown.* **What is New:** *• Performing immediate ventilation without intubation was associated with shorter—but not clinically relevant—time of initiation of ventilation compared to immediate laryngoscopy with intubation and suctioning in a manikin model.* *• Further studies in a clinical setting are warranted.*

**What is Known:**

*• Management of the non-vigorous newborn delivered through meconium-stained amniotic fluid remains still controversial.*

*• Tracheal suctioning in non-vigorous newborn delivered through meconium-stained amniotic fluid is supposed to delay initiation of positive pressure ventilation, but the magnitude of such delay is unknown.*

**What is New:**

*• Performing immediate ventilation without intubation was associated with shorter—but not clinically relevant—time of initiation of ventilation compared to immediate laryngoscopy with intubation and suctioning in a manikin model.*

*• Further studies in a clinical setting are warranted.*

## Introduction

Approximately 3–14% of deliveries are complicated by meconium-stained amniotic fluid (MSAF), which can cause meconium aspiration syndrome (MAS) in 5–10% of these newborns [1–3].

MAS has a multifactorial pathophysiology secondary to intrauterine asphyxia leading to chemical irritation, inflammatory response, surfactant inactivation, and airway obstruction [4–6]. MAS may result in several life-threatening complications (such as respiratory failure, pulmonary inflammation, persistent pulmonary hypertension, sepsis, neurological impairment, and chronic hypoxia) with a mortality rate of 20% in low-income countries [4, 7].

Although endotracheal suctioning can be used to clear a blocked airway, the International Liaison Committee on Resuscitation (ILCOR) suggests against routine tracheal intubation and suctioning for non-vigorous newborns delivered through MSAF [8]. Routine tracheal suctioning of such infants is likely to delay the initiation of positive pressure ventilation (PPV), especially when performed by inexperienced healthcare providers, hence possibly increasing the severity of hypoxic-ischemic encephalopathy [8]. However, the magnitude of the delay and its clinical relevance have not been measured so far. On the other hand, there is no evidence that routine tracheal suctioning provides clinical benefits when performed by experienced healthcare providers [8].

The present study aimed to compare the time of PPV initiation when performing immediate laryngoscopy with intubation and suctioning vs. performing immediate PPV without intubation in a manikin model.

## Methods

### Study design

This was a randomized controlled crossover (AB/BA) trial comparing PPV initiation with or without endotracheal suctioning in a manikin model simulating a non-vigorous neonate born through MSAF (clinicaltrial.gov NCT04076189). The AB/BA scheme is uniform within sequences and periods, thus removing any period and sequence effects [9]. The Ethics Committee of University of Padova (Italy) deemed that a formal ethical approval was not required since the study used manikin data (Prot. 0059234). Written informed consent was obtained from participants.

### Setting

This simulation study was performed at the University of Padua (Padua, Italy) between 16 and 24 September 2019. The scenario consisted of a non-vigorous newborn delivered through MSAF needing resuscitation (neonatal simulator manikin: Resusci Baby QCPR, Laerdal Medical, Stavanger, Norway). An external observer provided maternal history and verbal feedback during the scenario only if specifically required by the participant. A wall suction system set at 100 mmHg and equipment for airway management, including a 12-Fr suction catheter, endotracheal tube, and meconium suction device, were available and prepared before the start of each simulation. The procedure was performed using a C-MAC® video laryngoscope (Karl Storz GmbH & Co. KG, Tuttlingen, Germany).

### Participants

Level III NICU consultants (neonatologists) and pediatric residents trained in advanced airway management were eligible to participate in the study. Refusal to participate was the only exclusion criteria.

### Randomization

Participants were randomly assigned to AB or BA arms in a 1:1 ratio. Allocation was stratified for neonatologists and pediatric residents. Randomization was performed using a computer-generated random assignment list. Arm assignments were placed in sequentially numbered, sealed, opaque envelopes.

### Procedures

Participants in AB arm were assigned to perform the procedure with endotracheal suction, followed by the procedure without endotracheal suction. Participants in BA arm were assigned to the reverse sequence. A washout period of 6 h (one procedure in the morning and one in the afternoon) was included to reduce any carryover effects.

Before the study, participants were shown two videos demonstrating both procedures on a manikin.

During the procedure with endotracheal suction, participants were required to perform the following steps: (i) placing the baby under the infant warmer, (ii) oropharyngeal suctioning under vision with laryngoscope, (iii) orally endotracheal intubation, (iv) endotracheal suction with meconium suction device and then remove the endotracheal tube, and (v) re-intubation and starting positive pressure ventilation (PPV). An assistant was available to help participants in positioning the baby, holding the size 3.5 endotracheal tube and placing the suction device on the tube. Another assistant observed the intubation on the monitor of the video laryngoscope and confirmed correct positioning of the tube.

During the procedure without endotracheal suction, the participants were required to perform the following steps: (i) placing the baby under the infant warmer, (ii) drying the infant and stimulating to start breathing, (iii) oro- and nasopharyngeal suctioning, and (iv) starting PPV with a face mask.

During each simulation, an external observer collected the time of PPV initiation (as time elapsed from the moment when the baby was placed on the table to the start of PPV) using a stopwatch. The clock continued to run until participants achieved successful intubation and suction or effective PPV with face mask confirmed with the entering of the air in the manikin lungs and presence of chest movements.

All resuscitations were performed according to the international guidelines on neonatal resuscitation [10].

### Outcome measure

The primary outcome measure was the time of PPV initiation. There were no secondary outcome measures.

### Data collection

Randomization sequence, participant sex, participant experience, and time of PPV initiation were collected by an observer who was not involved in the simulation. Data were recorded on a data sheet designed for the study and stored in a password-protected computer to protect confidentiality before, during, and after the trial.

### Masking

Participants and outcome assessors could not be masked to treatment allocation due to the characteristics of the intervention. Participants were masked to the monitor of the video laryngoscope. The statistician who performed data analysis was masked to treatment allocation.

### Sample size

A minimum of 14 participants were required to have a 90% chance of detecting, as significant at the 5% level, a standardized effect size of 1 in a crossover design. The sample size was finally set at 20 participants (10 in AB arm and 10 in BA arm) for each strata (20 pediatric residents and 20 neonatologists).

### Statistical analysis

Continuous data were expressed as mean and standard deviation and categorical data as number and percentage. The study included a washout period that was chosen to reasonably prevent carryover effects. Timing of PPV initiation was compared between procedures (with vs. without endotracheal intubation and suctioning) using a paired Student’s *t* test. Period effects were tested using a two-sample Student’s *t* test applied to the differences between procedures [11]. The paired analysis was performed in pediatric residents and in neonatologists separately and was followed by the comparison of differences between procedures in pediatric residents vs. neonatologists (using a two-sample Student’s *t* test). Effect sizes were expressed as mean differences with 95% confidence intervals. All tests were two-sided, and a *p* value less than 0.05 was considered statistically significant. Statistical analysis was performed using R 3.5 (R Foundation for Statistical Computing, Vienna, Austria) [12].

## Results

**Fig. 1:**
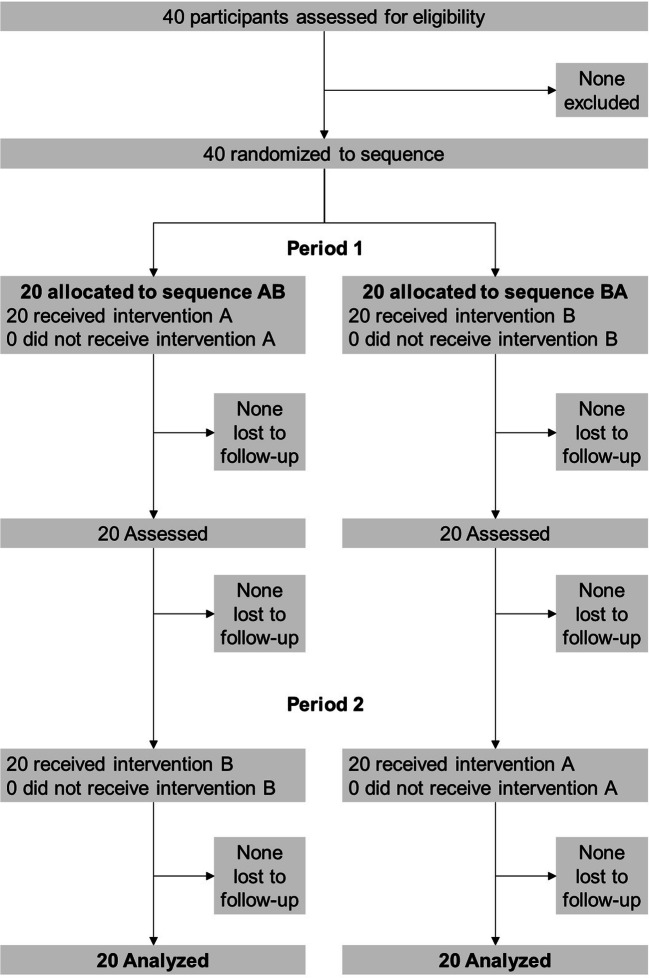
CONSORT flow diagram

The study included 20 pediatric residents (2 males and 18 females) and 20 neonatologists (5 males and 15 females) (Fig. 1). Experience in intubation was > 10 intubations in three residents, 5–10 intubations in eight residents, and < 5 intubations in 9 residents. All neonatologists had high experience in intubation. Successful intubation required two attempts in four pediatric residents and in one neonatologist, one attempt in the remaining participants.

Timing of PPV initiation in pediatric residents and neonatologists is shown in Table 1. In pediatric residents, timing of PPV initiation was longer with vs. without endotracheal intubation and suctioning (mean difference 13 s, 95% confidence interval 8 to 18 s; *p* < 0.0001). In neonatologists, timing of PPV initiation was longer with vs. without endotracheal intubation and suctioning (mean difference 12 s, 95% confidence interval 8 to 16 s; *p* < 0.0001). No period effect was found in pediatric residents (*p* = 0.36) or neonatologists (*p* = 0.24).

**Table 1:** Timing of PPV initiation (seconds)

	PPV initiation (s)				
	Procedure with endotracheal suction: mean (standard deviation)	Procedure without endotracheal suction: mean (standard deviation)	Mean difference (95% confidence interval)	*p* value (with vs. without endotracheal suction)	*p* value (pediatric residents vs. neonatologists)
Pediatric residents	43 (9)	30 (8)	13 (8 to 18)	< 0.0001	0.85
Neonatologists	38 (8)	26 (7)	12 (8 to 16)	< 0.0001	

The difference in timing of PPV initiation without vs. with endotracheal suctioning was similar between pediatric residents and neonatologists (mean difference − 1 s, 95% confidence interval − 7 to 6 s; *p* = 0.85).

Overall, all participants started PPV within 1 min as per ILCOR recommendations, but one pediatric resident with endotracheal suctioning (66 s).

## Discussion

Our findings indicate a statistically significant increase in time of PPV initiation with endotracheal intubation and suctioning compared with immediate PPV, but the difference does not seem clinically relevant. Such difference is comparable between low-experienced (pediatric residents) and experienced (neonatologists) health care providers.

To our knowledge, this is the first study comparing time of PPV initiation with vs. without endotracheal intubation and suctioning in a manikin. The strengths of the study include the crossover design, the use of a video laryngoscope to confirm the positioning of the tube in the trachea, and the inclusion of both inexperienced and experienced health care providers. These data can be used to inform the design of future trials in live newborns. The main limitation of the study is the use of a manikin. The drawbacks of using a manikin include the absence of meconium-stained fluids in upper airways (which can obstruct vision of the vocal cords), the lack of clinical feedback (i.e., bradycardia and hypoxia), and the lower stress environment. In addition, the inclusion of pediatric residents and neonatologists may limit the generalizability of the findings to settings with different resuscitation team.

Questions about the optimal management of the non-vigorous newborn delivered through MSAF remain unanswered [13, 14]. The most recent ILCOR consensus on science and treatment recommendation suggested against routine tracheal intubation and suctioning for non-vigorous newborns delivered through MSAF [8]. The rationale was based on unknown benefit of the intervention due to insufficient evidence to support it and harm avoidance due to potential delay in initiation of PPV and invasiveness of the procedure [8]. A systematic review including four RCTs found no difference in clinical outcomes (mortality and MAS) between approaches with or without tracheal suctioning [15–19]; however, no information on the timing of initiation of PPV was reported. Our study adds information on the magnitude of the delay in initiation of PPV due to tracheal intubation and suctioning. Such delay occurs, but it does not appear to be clinically relevant. Of note, all participants but one started PPV within 1 min as per ILCOR recommendations [8].

Although our finding suggests no harmful consequences due to delayed start of PPV, caution is suggested in generalization to clinical practice. According to previous studies on the intubation procedure [20, 21], it is reasonable to hypothesize longer times in live newborns compared with the manikin model. We cannot exclude that delayed start of PPV may affect oxygen saturation in live newborns, but this information could not be obtained from the manikin. The reader should also consider that the timing to perform intubation may be different when using a standard laryngoscope compared with video laryngoscope (which is not available in most of the delivery rooms around the world) [22, 23]. Finally, the experience of the operator may play an important role in the success and duration of the procedure [21]. Endotracheal intubation is a difficult procedure requiring trained staff and repeated practice to maintain adequate technical skills [21, 24]. Nonetheless, providing a correct and efficacious PPV with face mask also needs continuous training.

## Conclusions

Performing immediate laryngoscopy with intubation and suctioning was associated with longer—but not clinically relevant—time of initiation of PPV compared to immediate PPV without intubation in a manikin model. While suggesting negligible delay in starting PPV, further studies in a clinical setting are warranted.

## Abbreviations

ILCOR: International Liaison Committee on Resuscitation
MAS: Meconium aspiration syndrome
MSAF: Meconium-stained amniotic fluid
PPV: Positive pressure ventilation
